# Higher skeletal muscle mass may protect against ischemic stroke in community-dwelling adults without stroke and dementia: The PRESENT project

**DOI:** 10.1186/s12877-017-0433-4

**Published:** 2017-02-03

**Authors:** Yang-Ki Minn, Seung-Han Suk

**Affiliations:** 1 Department of Neurology, Hallym University, 1- shingil-ro, Yeoungdeungpo-Gu, Seoul, 07441 Republic of Korea; 2 Department of Neurology, Wonkwang University Sanbon Medical Center, Sanbon-Ro 321, Gunpo-si, Gyeonggi-do 15865 Republic of Korea; 3 Wonkwang University Ansan Municipal Geriatric Hospital and Center for Prevention of Stroke and Dementia, Ansan City, Gyeonggi-do Republic of Korea

**Keywords:** Sarcopenia, Body composition, Skeletal muscle mass, Risk factor, Stroke, Bioelectrical impedance analysis (BIA)

## Abstract

**Background:**

It is well known that a low skeletal muscle mass (SMM) is associated with stroke. However, it is unknown whether increasing muscle mass can prevent stroke.

**Methods:**

This community-based cross-sectional study was supported by the regional government. SMM measurements and brain computed tomography was performed in 722 stroke-free and dementia-free subjects (aged 50–75 years). Subjects were divided into quartiles (Q) by SMM, checked using the bioelectrical impedance analysis method (InBody 770, InBody, Seoul, Korea). Odds ratios (ORs) of brain white matter changes/silent infarction (WMC/SI) were calculated. The subjects were then divided into two groups by sex and evaluated.

**Results:**

In the analysis of the four groups, the unadjusted ORs of Q2–Q4 were 0.616 (95% confidence interval [CI], 0.372–1.022; *P* = 0.061), 0.290 (CI, 0.159–0.530; *P* < 0.001), and 0.209 (CI, 0.108–0.403; *P* < 0.001) for the risk of WMC/SI. Adjusted ORs for age, hypertension, diabetes mellitus, education, hypercholesterolemia, and smoking were 0.994 (CI, 0.513–1.740; *P* = 0.085), 0.669 (CI, 0.329–1.362; *P* = 0.268), and 0.464 (CI, 0.219–0.984; *P* = 0.045). In the two–group (dichotomized) analysis, the unadjusted OR for the higher muscle mass groups (Q3 + Q4) was 0.313 (CI, 0.200–0.491; *P* < 0.001). The adjusted OR was 0.577 (CI, 0.340–0.979; *P* = 0.042). Considering sex, the adjusted OR were 0.351 (CI, 0.141–0.869; *P* = 0.024) in men and 0.771 (CI, 0.391–1.519; *P* = 0.452) in women.

**Conclusions:**

Our findings suggest that increased SMM may protect against WMC/SI, especially in men.

## Background

Since Rosenberg first used the term “sarcopenia,” which refers to the involuntary loss of skeletal muscle mass (SMM) followed by strength [1, 2], it has been known as a risk factor for cardiovascular disease [3, 4].

We have shifted from the idea that sarcopenia is the risk factor for stroke to amount of skeletal muscle is preventive factor for stroke.

It is well known that chronic low grade inflammation cause atherosclerosis. The recent findings allow the skeletal muscle to be considered as an endocrine organ [5]. Contraction of skeletal muscle secret myokines and block the inflammatory signaling pathways generated by chronically elevated levels of pro-inflammatory adipokines [6] Muscle mass can be increased by weight training exercise. However, it is unknown whether increased muscle mass can prevent stroke. One study showed that increasing muscle strength in adolescent men had a decreased risk of later cardiovascular events in middle age [7]. Thus, we hypothesized that increased SMM can prevent stroke after middle age.

Stroke itself can cause sarcopenia [8, 9]. So skeletal muscle data after stroke might be contaminated by stroke itself. Thus, is important to collect skeletal muscle data before stroke occurs. Brain white matter changes/silent infarction (WMC/SI) represents an independent risk factor for cerebral infarction [10, 11]. Our study investigated the correlation between SMM and WMC/SI in a community-based setting.

## Methods

We have brought data from the Prevention of Stroke and Dementia (PRESENT) project, supported by the regional government that was initiated in July 2007. The primary goal of the PRESENT project is to prevent stroke and dementia using public education efforts, public relations, early medical check-ups, and research in Ansan City, Gyeonggi-do, Korea [12].

**Fig. 1:**
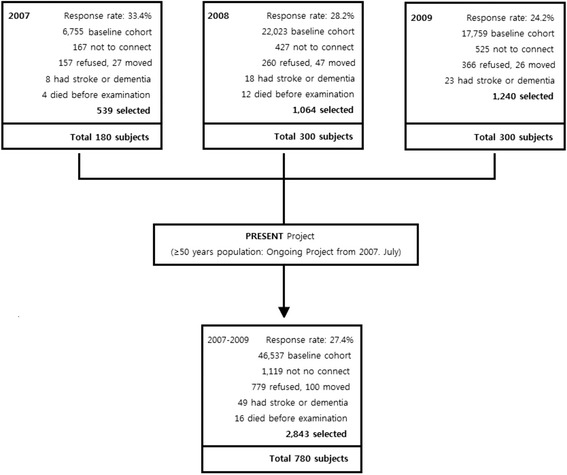
Flow chart of study participants

Stroke and dementia-free adults (aged 50–75 years) in Ansan City (The population of Ansan is 744,356; Dec.31,2016), were recruited between 2007 and 2009 by systemic random sampling using a previously described method (Fig. 1) [12]. All of the procedures were performed after written permission was obtained from each subject. Of the 780 subjects, we excluded those for whom some were missing (education level data; 54 subjects, SMM data; 4). Finally, 722 subjects were evaluated. Stroke history was screened by self-report and dementia was screened by mini mental state examination.

1. Risk factor assessment

Trained nurses and neurologists did personal interviews and evaluations Education, hypertension, smoking, diabetes mellitus, and hypercholesterolemia data were chyecked. Blood pressure was measured. Blood chemistry data were analysed. Hypertension was defined as a previous diagnosis of hypertension, the use of one or more anti-hypertensive drugs, a systolic pressure ≥ 140 mmHg, or a diastolic pressure ≥ 90 mmHg [13]. Hypercholesterolemia was defined as the use of one or more lipid lowering agents, a fasting total cholesterol level ≥ 240 mg/dL, a low-density lipoprotein cholesterol level ≥ 160 mg/dL, or a triglyceride level ≥ 200 mg/dL [14]. A participant was said to have diabetes mellitus if he/she had a previous diagnosis of diabetes mellitus, used anti-diabetic medication (including insulin), or had a fasting glucose level ≥ 126 mg/dL [15]. Smoking was defined as a current smoking habit.

2. Neuroimaging

We used a Brilliance™ CT Scanner (six slices; Philips, Eindhoven, the Netherlands) for brain computed tomography (CT). Two different neurologists blinded to the participants’ clinical conditions independently evaluated the results and laboratory findings as previously described [12]. The subjects were divided into the normal and WMC/SI groups according to the brain CT findings. The WMC/SI group included participants with WMC, SI, or both. SI was defined as well-defined areas > 2 mm showing attenuation without a relevant clinical neurological event. WMC was defined as ill-defined and moderately hypodense areas of >5 mm located in the periventricular or subcortical area, including extensive periventricular lesions and severe leukoencephalopathy [16, 17].

3. Measurement of skeletal muscle mass

SMM was measured by using an InBody720 body composition analyser (InBody, Seoul, Korea). The validity of this bioelectrical impedance analysis was documented in previous studies [18–20]. Subjects were divided into four quartiles (Q) based on their SMM using the 25th, 50th, and 75th percentiles. We separately applied the reference points because men tend to have greater SMM than women.

4. Data analyses

We used a multiple regression analysis to obtain odds ratios (ORs) [10] and unadjusted and adjusted ORs [21]. First, the independent variable was the four SMM quartiles. Well-known risk factors for WMC/SI, including age, hypertension, education, diabetes mellitus (DM), hyperlipidaemia, and current smoking status, were used as covariates. Next, the subjects were dichotomized into a high muscle mass group (HMMG, Q3 and Q4) and a low muscle mass group (LMMG, Q1 and Q2) because Q2 was not significant on univariate analysis when Q1 was used as a reference. Men and women were analysed separately. We used two-tailed *P* values for all analyses. The analyses were performed using SPSS for Windows, version 18 (SPSS Inc., Chicago, IL, USA). Significance was set at values of *P* < 0.05.

## Results

The baseline data for the 722 subjects enrolled in this study are summarized in Table 1. The subjects in the WMC/SI group were older, had a higher incidence of hypertension and DM, and had lower education levels compared to the normal brain CT group (Table 1). The mean SMM of each quartile is shown in Table 2.

**Table 1:** Risk factors for stroke among subjects in the normal and WMC/SI groups

	Normal (*N* = 613)	WMC/SI (*N* = 109)	*P* value
Mean age, years (SD)	58.5 (7.1)	68 (7.8)	0.000
Men, n (%)	276 (45)	45 (41.3)	0.469
Mean education, years (SD)	9.6 (4.4)	7.5 (5.1)	0.000
Hypertension, n (%)	307 (50.1)	94 (86.2)	0.000
Diabetes mellitus, n (%)	103 (16.8)	34 (36.7)	0.000
Hypercholesterolemia, n (%)	199 (32.5)	40 (36.7)	0.387
Current smoking, n (%)	106 (17.3)	19 (17.4)	0972
Mean skeletal muscle mass, kg (SD)	24.4 (9.6)	22.0 (5.0)	0.011
Skeletal muscle mass, Q			
Q1, n (%)	128 (20.9)	46 (42.2)	
Q2, n (%)	149 (24.3)	33 (30.3)	
Q3, n (%)	163 (26.2)	17 (15.6)	
Q4, N (5)	173 (28.2)	13 (11.9)

**Table 2:** Skeletal muscle mass by quartile

Quartile	Skeletal muscle mass, kg	
	Men	Women
1 (0–25th percentile)	<26	<18.3
2 (26th–50th percentile)	26–28.1	18.3–20
3 (51st–75th percentile)	28.1–30.7	20–21.6
4 (76th–100th percentile)	>30.7	>21.6

In normal CT subjects, the muscle mass groups were equally distributed (Q1 20.9%, Q2 24.3%, Q3 26.6%, Q4 28.2%, Q1 + Q2 45.2%, Q3 + Q4 54.8%). However, >70% of subjects in the WMC/SI group were in the lower muscle mass subgroup (Q1 42.2%, Q2 30.3%, Q3 15.6%, Q4 11.9%, Q1 + Q2 72.5%, Q3 + Q4 27.5%) (Table 2). In the quartile analysis, unadjusted analysis showed that the ORs for WMC/SI for each quartile (reference, Q1) were 0.616 (95% confidence interval [CI], 0.372–1.022, *P* = 0.061) in Q2, 0.029 (95% CI, 0.159–0. 53; *P* < 0.001) in Q3 and 0.209 (95% CI, 0.108–0.403; *P* < 0.001) in Q4. After adjustment for age, hypertension, DM, education, hypercholesterolemia, and smoking, the ORs for WMC/SI for each quartile (reference, Q1) were 0.944 (95% CI, 0.513–1.742; *P* =0.854) in Q2, 0.669 (95% CI 0.329–1.362, *P* = 0.268) in Q3 and 0.046 (95% CI, 0.219–0.984; *P* = 0.045) in Q4.

**Fig. 2:**
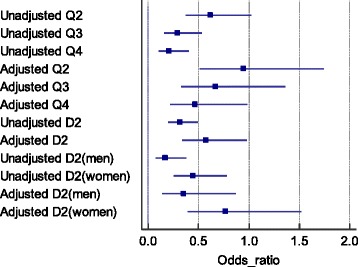
Odds ratios of the each muscle mass group for white matter change/silent infarction on brain computed tomography (reference, quartile 1 [Q1] or D1). Adjusted for age, education, hypertension, diabetes mellitus, and current smoking. Q1, 1st-25th percentile group; Q2, 26th-50th percentile group; Q3, 51st-75th percentile group; Q4, 76th-100th percentile group; D1, 1st -50th percentile group; D2, 51st-100th percentile group

In the dichotomized analysis, the unadjusted analysis showed that the ORs for WMC/SI for the HMMG were 0.313 (95% CI, 0.200–0.491; *P* < 0.001). After adjustment for age, hypertension, DM, education, hypercholesterolemia, and smoking, the ORs for WMC/SI for HMMG were 0.577 (95% CI, 0.341–0.979; *P* = 0.042). After men and women were divided by sex, the adjustment ORs were 0.351 (95% CI, 0.141–0.0869; *P* = 0.024) in men and 0.771 (95% CI, 0.391–1.519; *P* = 0.452) in women (Fig. 2).

## Discussion

Long term exercise prevents cardiovascular risk factor by secret various anti-inflammatory myokine by skeletal muscle [22]. There are two ways to increasing anti-inflammatory myokines by skeletal muscle, increasing physical activity and increasing muscle mass.

Our data showed that individuals in the high HMMG displayed fewer WMC/SI areas on the brain CT. Considering that WMC/SI of the brain is closely correlated with further cerebral infarction [10, 11], we can hypothesize that men in the HMMG had a lower risk of cerebral infarction. Furthermore, we hypothesize that increasing SMM by exercise maybe associated with protection of cerebrovascular dieseases.

In the unadjusted and adjusted analyses, we detected a trend of a protective effect of higher SMM for cerebral vascular lesions. The dichotomized analysis also showed a significant protective effect on HMMG. When we divided the subjects by sex, the protective effect of a high SMM against stroke was observed only in men (Fig. 2). Because diabetes mellitus, hypertension, hypercholesterolemia and SMM, there are difference in data before and after adjustment.

The protective effect of increased SMM against stroke risk was apparent in men. We cannot explain this phenomenon; a previous study also demonstrated the protective effect of muscle strength only in men [7]. Although women did not demonstrate a negative effect of increased muscle mass, they were excluded from that study. We believe that this is due to the difference in muscle composition ratios. It is known that many muscle parameters such as fat free mass, SMM, and skeletal muscle index are related to sex. Or absolute HSMG has protective effect. Men in Q1 had a higher absolute SMM than women in Q4 (Table 2).

An individual’s SMM peaks at 24 years of age and is maintained quite well throughout the fifth decade with a moderate decline of about 10% [23]. The SMM then decreases up to 40% between ages 50 and 80 years [24]. Therefore, we recruited subjects > 50 years of age for the current study.

No gold standard exists to assess SMM. Dual-energy X-ray absorptiometry (DEXA) can be used to assess SMM [25], but a DEXA scanner is a fixed and expensive piece of equipment that utilizes ionized radiation. By comparison, eight-polar bioelectrical impedance analysis (BIA, using the InBody720, for example) is a simple and inexpensive method whose results correlate well with those of DEXA [20]. High SMM may secondary effect of long term exercise. But this fact is not important. In BIA era, SMM is simple, repeatable and objective biomarker.

We did not use an index that divided SMM by other parameters such as height or body surface area. It would be appropriate to use such an index rather than an absolute value. However, Rantandem et al. demonstrated that muscle strength itself was an independent risk factor of mortality regardless of body mass index [26]. Height is used in an algorithm for calculating SMM by bioelectrical impedance analysis. The exact algorithm used by the InBody system is not disclosed. Therefore, dividing SMM by height is not appropriate.

According to the current guidelines for the primary prevention of stroke, obesity is a well-known modifiable risk factor for stroke. However, in the previous versions of the guideline available when the PRESENT project was designed, obesity was a less well-documented risk factor. Therefore, we did not include obesity data in this study [21]. This is a limitation of our study.

This was a cross-sectional rather than prospective study. We included stroke- and dementia-free subjects who could visit city health centers by walking. A brain CT was performed at the same time as the SMM assessment. We believed that the study’s cross-sectional design could eliminate the long-term indirect effect of exercise. Preserved muscle fitness in obesity may not only prevent sarcopaenic obesity but also decrease associated risks for metabolic syndrome and early mortality [27].

We do not measure inflammatory cytokine such as IL-6,10. This is one of major limitation of this study.

## Conclusions

This study findings suggest that increased SMM may be associated with a protective effect against WMC/SI in community-dwelling men without stroke or dementia. Regular weight training exercises to increase SMM may help prevent stroke.

## Abbreviations

CT: Computed tomography
DEXA: Dual-energy X-ray absorptiometry
DM: Diabetes mellitus
HMMG: High muscle mass group
LMMG: Low muscle mass group
PRESENT: Prevention of stroke and dementia
Q: Quartiles
SI: Silent infarction
SMM: Skeletal muscle mass
WMC: White matter changes
